# The Smartphone-Based Offline Indoor Location Competition at IPIN 2016: Analysis and Future Work

**DOI:** 10.3390/s17030557

**Published:** 2017-03-10

**Authors:** Joaquín Torres-Sospedra, Antonio R. Jiménez, Stefan Knauth, Adriano Moreira, Yair Beer, Toni Fetzer, Viet-Cuong Ta, Raul Montoliu, Fernando Seco, Germán M. Mendoza-Silva, Oscar Belmonte, Athanasios Koukofikis, Maria João Nicolau, António Costa, Filipe Meneses, Frank Ebner, Frank Deinzer, Dominique Vaufreydaz, Trung-Kien Dao, Eric Castelli

**Affiliations:** 1 Institute of New Imaging Technologies, Universitat Jaume I, 12071 Castelló, Spain; montoliu@uji.es (R.M.); gmendoza@uji.es (G.M.M.-S.); belfern@uji.es (O.B.); 2 Centre for Automation and Robotics (CAR), CSIC-UPM, 28500 Arganda del Rey, Spain; antonio.jimenez@csic.es (A.R.J.); fernando.seco@csic.es (F.S.); 3 Faculty for Geomatics, Computer Science and Mathematics, HFT Stuttgart—University of Applied Sciences, 70174 Stuttgart, Germany; stefan.knauth@hft-stuttgart.de (S.K.); athanasios.koukofikis@hft-stuttgart.de (A.K.); 4 Algoritmi Research Centre, University of Minho, 4800-058 Guimarães, Portugal; adriano.moreira@algoritmi.uminho.pt (A.M.); joao.nicolau@algoritmi.uminho.pt (M.J.N.); antonio.costa@algoritmi.uminho.pt (A.C.); 5 BlockDox, 129 Finchley Road, NW3 6HY London, UK; yaia1309@gmail.com; 6 Faculty of Computer Science and Business Information Systems, University of Applied Sciences Würzburg-Schweinfurt, 97070 Würzburg, Germany; toni.fetzer@fhws.de (T.F.); frank.ebner@fhws.de (F.E.); frank.deinzer@fhws.de (F.D.); 7 MICA Institute (HUST-CNRS/UMI2954-Grenoble INP), Hanoi University of Science and Technology, 100000 Hanoi, Vietnam; viet-cuong.ta@inria.fr (V.-C.T.); trung-kien.dao@mica.edu.vn (T.-K.D.); eric.castelli@mica.edu.vn (E.C.); 8 Centro de Computação Gráfica (CCG), 4800-058 Guimarães, Portugal; filipe.meneses@ccg.pt; 9 Pervasive Interaction/LIG, CNRS, Université Grenoble Alpes, Inria, LIG, F-38000 Grenoble, France; Dominique.Vaufreydaz@inria.fr

**Keywords:** indoor localization technology, indoor navigation, smartphone applications, evaluation and benchmarking

## Abstract

This paper presents the analysis and discussion of the off-site localization competition track, which took place during the Seventh International Conference on Indoor Positioning and Indoor Navigation (IPIN 2016). Five international teams proposed different strategies for smartphone-based indoor positioning using the same reference data. The competitors were provided with several smartphone-collected signal datasets, some of which were used for training (known trajectories), and others for evaluating (unknown trajectories). The competition permits a coherent evaluation method of the competitors’ estimations, where inside information to fine-tune their systems is not offered, and thus provides, in our opinion, a good starting point to introduce a fair comparison between the smartphone-based systems found in the literature. The methodology, experience, feedback from competitors and future working lines are described.

## 1. Introduction

Personal indoor localization is still an open problem, for which many different approaches and technologies have been proposed, with the aim of obtaining a usability similar to that achieved outdoors by the GPS system [1,2,3]. Some indoor positioning techniques are based on specifically designed beacons for localization, while others restrict themselves to the already existing infrastructure in buildings, such as Wi-Fi Access Points (APs), or other so-called signals of opportunity (magnetic field, pressure, light and sound intensity, Global Navigation Satellite Systems (GNSS), among others). New challenging applications, such as ubiquitous indoor positioning (e.g., [4]), may be enabled by the data generated by smartphone sensors and the crowd’s movement [5,6].

Many indoor positioning designs found in the literature are accompanied by physical experimentation and evaluation of the system (about 77% of works, according to [7]). However, most of these evaluations are carried out in rather restricted areas; usually, a few university departments easily accessible to the researcher [8]. A second related problem is that the raw data collected and used during the evaluation of the positioning system is not made available to the research community, making it impossible to confirm the claimed results. Both issues make a critical comparison of Indoor Positioning Systems (IPS) developed by different groups not feasible in a rigorous manner, since collected signals, context and scenarios may change in an uncontrolled way.

A better way to compare positioning algorithms under the same experimental conditions would be using a repository of prerecorded data in a large variety of buildings and contexts. An inspiration for this approach is the Machine Learning Repository of the University of California Irvine (UCI) [9] and Kaggle [10], both created for evaluating machine learning algorithms with common databases. Although some related databases [11,12,13,14,15,16,17,18,19,20,21,22], interesting initiatives for benchmarking [23] and even competitions (Microsoft-IPSN [24,25,26,27], EvAAL [28,29,30,31,32] and EVARILOS [33,34]) for better evaluation of IPS already exist, no one encompasses the wide diversity in technologies, contexts and scenarios currently found in this field.

A competition is a convenient way to initiate IPS comparisons and reach a worldwide recognition of datasets for fair evaluation. The first off-site indoor location competition was the EvAAL-ETRI Indoor Location [35,36], which was held during the Sixth International Conference on Indoor Positioning and Indoor Navigation (IPIN 2015). The third track, called “Wi-Fi fingerprinting in large environments” was off-site, and the competitors had access to a large Wi-Fi fingerprint database, the UJIIndoorLoc [15], to which they had to apply their positioning estimation approaches offline. In the current edition, the organizers decided to make the off-site competition more challenging by creating a new database including the data provided by all sensors embedded in typical smartphones, acquired by different people moving in different types of buildings.

The aim of this paper is to contribute to the definition and creation of a new framework for the evaluation of smartphone-based IPS based on our experience at the IPIN2016 off-site competition. We will:

1. describe the database and the evaluation criteria used
2. analyze and compare the competing IPS under equal evaluation conditions
3. present the experiences and suggestions from the competitors to enhance the evaluation framework
4. discuss directions to improve a repository that could be used as a universal reference for testing smartphone-based IPS

The remaining of this paper is organized as follows. Section 2 describes the IPIN 2016 off-site competition track. Section 3 introduces the competing teams and their competing systems. Section 4 shows the results of the competition and their analysis. Section 5 contains the discussion about the current competition and the future lines to improve it. Finally, the conclusions are given in Section 6.

## 2. The IPIN 2016 Off-Site Competition

The goal of the competition was to evaluate the performance of different indoor localization solutions based on the signals available in a smartphone (not augmented by any additional hardware) and received while a person was walking in a realistic multi-building, multi-floor indoor environment.

Any kind of positioning estimation method based on the provided data was admitted to the competition, for example:

- Wi-Fi or magnetic-based fingerprinting.
- Multi-sensor fusion algorithms trying to exploit dynamic time-correlated information.
- Innovative approaches using map information or activity recognition to complement the above-mentioned ones.

This section introduces the main features of the off-site competition, the description of the data provided to competitors and the evaluation criteria.

### 2.1. Main Features of the Competition

**Figure 1:**
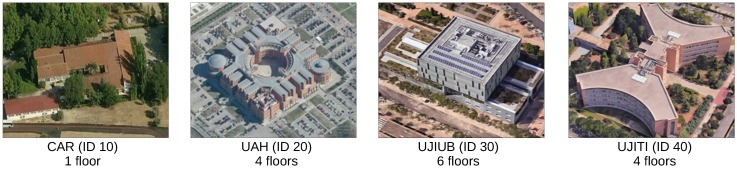
Satellite view of the four buildings used in the Indoor Positioning and Indoor Navigation (IPIN) 2016 competition. The building identifiers are also included in the figure (see Section 2.3).

The main features of the competition were:

- *Off-site competition approach*. This track was done off-site and offline, so all data processing for calibration and evaluation had to be done before the date of the IPIN conference. Competitors were provided with sensor data logfiles acquired with different mobile phones, including ground truth (known trajectories), that could be used by the teams for tuning their models, as well as data for evaluation (all sensors’ data without ground truth, unknown trajectories).
- *Multiple sources of information*. The provided data logfiles were captured by using several conventional modern smartphones and a dedicated Android application named GetSensorData [37]. The logfiles contained all of the available signals that were captured in real time with a smartphone: Wi-Fi Received Signal Strengh (RSS), inertial data, magnetic field, GPS, and pressure, among others.
- *Continuous motion and recording process*. While recording the logfiles with the smartphone, the person moved along a continuous trajectory passing by some known landmarks that were recorded in the logfiles.
- *Realistic walking style*. The person recording the data moved in a natural way: most of the time walking forward at normal speed, but occasionally taking 90 or 180 degree turns (e.g., at corridor ends), moving backward or laterally at certain points (e.g., if giving way at door accesses), changing floors through elevators and stairs, etc. The user speed was approximately constant when recording the data with eventual stops at some positions.
- *Phone holding*. The phone was hand held at all times by the user, either stable in front of his face or chest (typical position for reading or typing with the phone), or with the arm downwards while holding the phone in his hand (producing a natural arm swing if walking). No pocket use, calling or any strange handling conditions were considered while collecting data.
- *Realistic environment and diversity* The competition took place in four different buildings (see Figure 1) that were not modified by installing any additional hardware. Moreover, different smartphones were used to gather the data so the competition was not attached to the features of any particular smartphone.

With respect to the 2015 EvAAL-ETRI off-site competition, the competition organizers introduced several major changes, with the goal of improvement of the indoor location systems’ performance and increasing the interest for the competition itself. Those changes were:

- Data came from multiple sensors in 2016 competition.
- Database is now provided in logfiles, as sequences of readings from multiple sensors.
- Data has been gathered while the user is moving, whereas data were statically captured in 2015.
- The reference database is not explicitly divided into training and validation sets , including only data with ground truth and data without ground truth. Data without ground truth was used for the evaluation of the different IPS.
- Additional information about the reference dataset was provided: floorplan maps, map-based reference trajectories and videos.
- The testing scenario is comprised of heterogeneous buildings at very different locations.

### 2.2. Testing Buildings

The competition environment comprised a total of four buildings. One of the buildings was the venue of the on-site IPIN 2016 Conference competition, the Polytechnic School at the University of Alcalá (UAH), Alcalá de Henares, Spain. In order to avoid possible interference between the on-site and off-site tracks, they took place in non-overlapping sectors of the building. The other three evaluation buildings (see Figure 1) correspond to CAR (CSIC Arganda, Madrid, Spain), UJIUB (Universitat Jaume I, Castellón, Spain) and UJITI (Universitat Jaume I, Castellón, Spain).

### 2.3. Description of Datasets (Logfiles)

**Figure 2:**
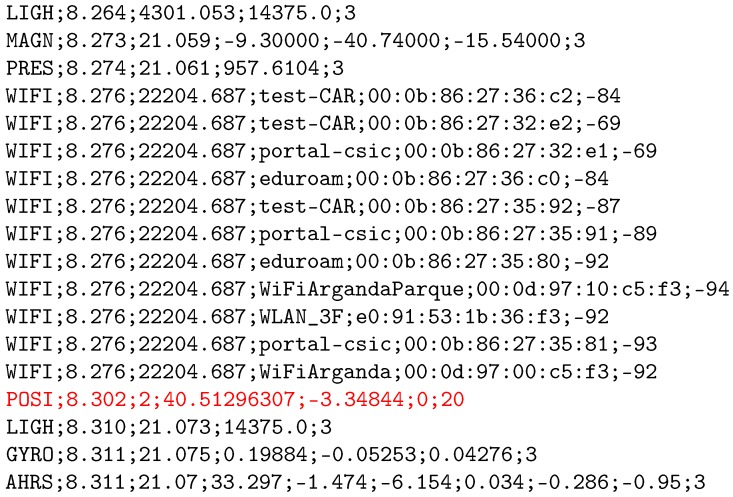
An excerpt from a logfile, as provided to the competitors. Note that the example includes a POSI entry (ground truth location highlighted in red color in the excerpt), but these entries are not included in the evaluation files since they do not contain ground truth locations.

The stream of sensor data generated in the phone is sequentially registered at the time it is obtained in a plain text file (called a *logfile*). Each line in the logfile corresponds to a single sensor reading, beginning with an initial header identifier (such as WIFI, ACC, or MAGN, among others) that determines the kind of sensor reading, and several fields separated by semicolons with different values. Additionally, those lines starting with the POSI header identifier does not belong to a sensor reading and they contain the ground truth location provided by the users. A parser in Matlab code (version 1, CSIC, Arganda del Rey, Madrid, Spain) [37] was provided to competitors in order to facilitate data rearrangement in the preferred format. Figure 2 shows an excerpt of a logfile.

The sampling rate of each sensor varied from logfile to logfile, depending on the embedded sensor chips used by a particular phone and version of the operating system. Typical sampling frequency values for the inertial data is about 50 Hz, while pressure, sound and light sensors have a much lower update rate (<10 Hz). Wi-Fi scans are available every 4.7 s in average according to the analysis done by the UMinho team. Wi-Fi scans depend on the device and they are generally available every 4 to 6 s (0.25 to 0.17 Hz).

Although multiple Wi-Fi access points were registered in the logfiles, their positions were unknown to the competitors. This is to emulate real world cases, where the positions of the access points is often unavailable. Nevertheless, several geo-referenced floor-map images for each building and some videos were provided to the competitors as additional data.

#### Dataset Types

As mentioned earlier, logfiles provided to the competitors were divided into training (known trajectories) and evaluation (unknown trajectories) logfiles. The training logfiles contained geo-referenced positions at some landmarks, in the form of additional records with a POSI header followed by the ground truth coordinates: latitude, longitude, floor and building identifiers (see highlighted line in Figure 2). The latitude and longitude coordinates are in WGS84 format, the floor corresponds to the floor number, and the building identifiers were introduced in Figure 1.

The competition organizers published the training logfiles in the official website, including geo-referenced positions at some landmarks. On 25 July, the organizers provided the evaluation logfiles to the competitors, which contained measurements taken following the same procedure of the training files (although possibly by different users or phone models), but without geo-referenced positions (POSI data). Thus, the competitors had to provide their estimations of the unknown trajectories by using only the phone sensor data without any landmark information.

Table 1 and Table 2 show a summary of the training and evaluation logfiles. Experimental diversity comes from the variation of trajectories, buildings, users and devices used to collect data.

### 2.4. Submission of Results and Evaluation

After processing the evaluation logfiles, participants had the opportunity to submit up to three different sets of indoor location estimates. The estimates had to be provided at a pace of 0.5 s and starting from the first timestamp available in each logfile. Each submission had to be independently provided to the organizers for its evaluation. The estimations had to adhere to the following format: timestamp, latitude, longitude, floor, and building.

Each submission was evaluated considering the estimates provided for the nine evaluation logfiles. The base positioning error was defined as the geometric distance between the two-dimensional (latitude and longitude) real position as recorded by competition organizers and the estimated position provided by the competing IPS. Furthermore, we took into account floor and building mis-identifications during the trajectories. In this case, 15 and 50 meter penalties were added to the geometric error if the competitors did not guess the correct floor and building, respectively. The final metric used for the competition results was the third quartile (75%) of this joint positioning error.

Of the three alternative sets of estimations submitted by the competition teams to the final tests, only the one with the best results was considered for the ranking of competitors.

## 3. Description of the Competing IPS

This section highlights the main features of the competing teams and references their work in the IPIN 2016 proceedings:

- The *HFTS team*: S. Knauth and A. Koukofikis. Stuttgart University of Applied Sciences, Stuttgart, Germany [38].
- The *UMinho team*: A. Moreira, M.J. Nicolau, A. Costa and F. Meneses. University of Minho and Centro de Computação Gráfica, Guimarães, Portugal [39]
- The *BlockDox (BD) team*: Y. Beer. BlockDox, London, United Kingdom (This competing team did not submit a paper to the conference).
- The *FHWS team*: T. Fetzer, F. Ebner and F. Deinzer. University of Applied Sciences Würzburg-Schweinfurt, Würzburg, Germany [40].
- The *Marauder team*: V.C. Ta, D. Vaufreydaz, T.K. Dao, and E. Castelli. Université Grenoble Alpes, CNRS, Inria, LIG, Grenoble, France and Hanoi University of Science and Technology, Hanoi, Vietnam [41].

### 3.1. The HFTS Team

The HFTS system employed GNSS, Wi-Fi, accelerometer, compass, and gyroscope data [42]. Accelerometer data was used to detect step events, and compass and gyroscope were used for heading estimation. Drift compensation and step length estimation were performed by a particle filter using the information of floor plans to detect the most likely path (see also [43,44]). Floor detection was based on position and received signal strength indicator (RSSI) evaluation. For the RSSI positioning, the scalar product correlation fingerprinting algorithm [45] was applied.

Heading detection was performed by combining gyro and magnetic information: the gyroscope is able to detect heading changes quite accurately on a short timescale, but will drift in the long-term. Therefore, the algorithm stabilized the gyro heading with the compass heading. Compass heading is subject to strong local magnetic perturbations but shows no drift on a long-term scale. The estimated heading was calculated by summing gyro heading changes immediately, but relaxing back to magnetic heading with a configurable constant.

Step detection was obtained by filtering the accelerometer data and detecting maximal and minimal values of the result. Each time a step was detected, the particle filter updated its state. The particle filter contained a constant number of particles. Besides the position, a particle state also comprised individual step length and heading offset values.

On each step, all particles were moved according to the estimated heading, individually modified by the particle specific offsets. Each time a particle collided with, for example, a wall, it was replaced by a new one. For collision detection, the provided floor plans were used. This new particle was seeded at the position of an existing particle, but had its own step length and heading offset value. The new seeded particle data contained also a reference to the particle where it was created.

A particular feature of the algorithm was its backtracking capability. By recursively tracking back the history of a particle, a continuous track estimation for each individual particle was possible, allowing a posteriori elimination of failed particles. This could lead to a significant accuracy increase for cases where the position information was not needed in real time. This method is related to particle backward smoothers (for example [46]), as well as loop closing.

### 3.2. The UMinho Team

The UMinho approach is mostly based on Wi-Fi fingerprinting enhanced with information extracted from the accelerometers and atmospheric pressure data. Thus, the UMinho team used only the POSI, Wi-Fi, accelerometer and atmospheric pressure records, ignoring the remaining data. The strategy involved three steps: creation of additional POSI records to increase the ground truth information density, segmentation of the data in periods of movement and immobility, and association between positions and Wi-Fi fingerprints.

Building the radio map raises a few challenges. The POSI records are sparse in time and the user movement cannot be modelled by straight lines between adjacent POSI records since they involve crossing walls or floors in the provided building blueprints. Thus, intermediate POSI records were created based on the videos of the data collection process, or, in cases where video was not available, by our best guesses of the most likely path compatible with the building blueprints. With the new additional records, the sequence of POSI records represents the estimated real trajectory.

The user movement was obtained from the accelerometer readings. The team removed the DC component of the acceleration magnitude signal and applied a one-dimensional median filter and a Butterworth filter to clean the data. Using the peaks of the resulting data, it was possible to count the steps and step times and determine if the user was moving or not.

As previously mentioned, the UMinho team approach is mainly based on the Wi-Fi fingerprint data; this requires building a Wi-Fi radio map, where each Wi-Fi fingerprint has to be associated to a geographic position. The entire set of fingerprints was used to create a radio map. Since Wi-Fi records were not created simultaneously with the POSI records, different strategies were tried to identify the coordinates for each fingerprint. In the end, the radio map was built by associating fingerprints to the linearly interpolated coordinates between known positions, combined with the movement information extracted from the accelerometer data.

Once the radio map is built, it is possible to estimate the trajectories in each of the evaluation dataset. For each fingerprint in the evaluation dataset, it was necessary to estimate the corresponding location. To estimate the building, a majority rule was used. To estimate the floor, the team used a filtering function combined with another majority rule [47]. Additionally, the pressure data was also used to estimate the floor. Having estimated the building and the floor, a k-Nearest Neighbors (kNN) [48] based method was used to estimate the coordinates using the fingerprints contained in the radio map that are most similar to the one being processed [49].

With the previously described method, the UMinho team was able to estimate the position (building, floor and coordinates) for the provided evaluation datasets. However, Wi-Fi fingerprints were collected with an average sampling period of 4.7 s and, as defined in the competition rules, the final trajectory estimation must be provided at discrete time intervals of 0.5 s. For this purpose, different strategies were considered, based on using linear interpolation and the mobility profile along each trajectory built from the accelerometer data. In the end, with these different strategies, three different solutions were built [39].

### 3.3. The BlockDox Team

The algorithm proposed by the BD team was on Random Forest fingerprinting assisted by GPS in order to detect outdoor location. Location was solved hierarchically in order to reduce the number of features in each step. Finding the correct building, then the floor and finally the location, all steps are done by using Wi-Fi fingerprinting. The first step was to associate a ground truth to the sensors’ records. The sensors’ records were not synchronized with the ground truth points. In order to associate the sensors’ records with ground truth, linear interpolation was used between ground truth locations and the floor and building identifiers of the point with the closest time. Wi-Fi fingerprinting only relays on power without a direction of arrival; therefore, this method depends on different Radio Frequency (RF) blockage from different Wi-Fi APs in order to get distinct fingerprints for different cells. Change in loss due to distance is hard to determine due to reflections. Using it in large halls or outside the building could lead to inaccuracy. GPS location was used to complete the Wi-Fi fingerprinting method.

#### 3.3.1. Hierarchical Algorithm

The primary classification was the building classification. The media access control (MAC) addresses of the visible Wi-Fi access points were orthogonal between buildings; therefore, it was possible to count known MAC addresses from each building and reducing the number of features from 730 unique addresses to four counters of known addresses, one per building, getting 100% correct results.

The secondary classification was floor estimation. The MAC addresses were not orthogonal between floors, and the station receives access points from multiple floors. MAC address fingerprinting was used to predict the floor because it is a finer approach and the number of MAC addresses is reduced. The final classification was based on Wi-Fi fingerprinting.

The classification used only the MAC addresses of the already predicted building and floor. The tested area was split into multiple cells and Wi-Fi RSSI features were used in a Random Forest Classifier algorithm in order to decide in which cell the device is located in. Time series attributes were applied in order to filter bogus predicted locations and smooth out the result. It allowed a drastic reduction in features.

#### 3.3.2. Cross Validation and Optimization

The training files were divided into different routes. To avoid overfitting, each route was evaluated using the other routes for training. The algorithm hyper parameters were hierarchically and greedily optimized in order to reduce the complexity and time of solving the problem—first optimizing the parameters of the floor classification, and only then the location parameters. When the optimum of each parameter was found, it was turned into a constraint and the optimization procedure continued to optimize the next parameter.

### 3.4. The FHWS Team

The FHWS localization system is based on previous works, primarily on the approach presented in [50]. Since then, this technique has been extended by including prior navigation knowledge using realistic human walking paths [51] and adding smoothing methods [40]. Additionally, a self-developed map editor allows for creating advanced 3D maps and realistically shaped stairs. This proposal avoids time-consuming fingerprinting and calibration processes. Furthermore, it does not need any prior information on the starting position. The system has been implemented in C++ using the Qt cross-platform application framework.

The smartphone provides all necessary measurements and no additional devices are needed. The readings of all smartphone sensors are fused using recursive density estimation, implemented by a particle filter with the state transition as proposal density. This transition is based on random walks along a 20 cm-grid graph, derived from the building’s floorplan. By removing edges intersecting with walls, it is ensured that only valid movements can be sampled. The random walk along the graph’s edges is constrained by the measured heading change and detected number of steps. Additionally, each node within the graph contains a weight, denoting the likelihood of it being visited by the pedestrian. Using this approach, nodes near walls receive a lower likelihood, while nodes marked as staircases, elevators and doors are more likely. During the state transition, this importance is used to increase the weight between two nodes.

The probability density of the state evaluation is provided by two probabilistic sensor models. First, the smartphone’s barometer, if available, is used to infer the likeliness of the current *z* location. As ambient pressure is dependent on environmental conditions like weather, time of day and others [50], relative pressure values were used instead of absolute ones. Due to noisy sensors, multiple readings are used to estimate this relative base. A probability is then obtained by comparing the measured relative pressure with a pressure prediction. This relative prediction is adjusted whenever a *z* change happens within the transition. Secondly, the Wi-Fi component provides absolute location estimates by measuring the RSS of nearby APs and comparing them with the expected signal strengths, determined with a wall attenuation factor model [50]. Thus, no fingerprinting is required. To reduce the setup time, the same parameters are used for all transmitters at the expense of a worse location estimation performance and higher uncertainty. Since the AP positions were not provided, a genetic algorithm is used to approximate the model based upon the RSS measured at the ground truth positions.

Finally, a fixed interval smoother, using backward simulation, is deployed for further optimisation and to reduce multimodalities [40]. Here, a smoothing transition model compares the distance, angle and height between some future and the current state. The resulting likelihood is then used for reweighting the particles.

### 3.5. The Marauder Team

The Maraduder team methodology for solving the challenge is to try as many indoor positioning methods as possible. The methods were evaluated by using a cross-validation scheme on the provided data. Based on the observation, several adjustment were applied in order to decrease the overall distance errors.

Building identification was trivial with the GNSS and Wi-Fi data. The approach started with using Wi-Fi data for classifying floor and inferring positioning. Generally, the two problems have the same feature space. They only differ in the learning targets. The former is to learn the floor number directly and the latter is to learn the 2D positioning.

#### 3.5.1. Floor Identification and Inferring Absolute Position

From the provided training data, a grouping step was applied to get the Wi-Fi fingerprinting database. Two notable characteristics of the resulting data were its sparseness in terms of the feature space (most of the APs have unseen value) and in terms of sampling points.

For the learning task, three models were applied: k-Nearest Neighbour, Random Forest [52] and Extreme Gradient Boost [53]. In addition, the set of features was varied: raw RSS value features, K-Filtering [49], and Hyperbolic Location Fingerprinting features [54]. Each model was tested with classifier and regressor.

#### 3.5.2. Path Approximation within the Floor

For path reconstruction within the floor, it was necessary to estimate the moving distance and direction. By applying a threshold on the standard deviation of accelerometer values, it is possible to recognize whether the user is standing or walking. In the case of walking, the moving distance was calculated based on the average speed, assuming it did not change throughout the logfile.

The user moving direction was assumed to coincide with that of the phone, as derived from accelerometer, magnetic and gyroscope information. Several ways of extracting the phone’s direction were tested, including using rotation matrix from magnetic and accelerometer, using gyroscope integration, Madgwick filter [55] and AHRS data directly.

From all of the approaches above, the value of Azimuth (or yaw) axis was used as the user’s direction of movement. A particle filter was used to reconstruct the full path. The particle only included a moving model with the direction and moving distance. A working observation model based on a Wi-Fi fingerprint model could not be created. To reduce the drifting errors, Wi-Fi data and map information were integrated into the approximating path.

Instead of building an observation model for each particle, a method which adjusts the particle on the basis of a spatial probability was proposed. The WiFi feature vector at time *t* was denoted by *X*, and *Y* was the position estimated by the Wi-Fi fingerprinting model, a particle *P* then could be pulled to position $P_{\mathrm{new}}$ closer to *M* with a constant *λ* as $P_{\mathrm{new}}=P+\lambda \times \left(Y-P\right)$. The position *Y* came from the prediction of Wi-Fi fingerprinting and the position of the three closest centers to *P*.

To avoid hitting walls, the movement of each particle was constrained. When a particle went across a wall, its direction was adjusted to go parallel with the wall. This approach could be efficient to stabilize the errors in a short term. On the other hand, its performance depended on the properties of the area where the user is located.

An optimization problem, with two operators, was defined to adjust the path and minimize the number of wall crossings. The first operator adjusts the speed if the user goes along a straight line, since the movement could be sometimes faster or slower than the average speed; the second operator adjusts the angle when the user turns, as the angle from the four methods above is unstable. To find a proper solution to this optimization problem, a greedy strategy was employed. From the starting configuration, the first collision with walls was detected. A grid search for the speed and angle adjusting was carried out. The range of grid should reflect the standard deviation errors in the process of inferring moving speed and direction.

## 4. Analysis of Results

**Figure 3:**
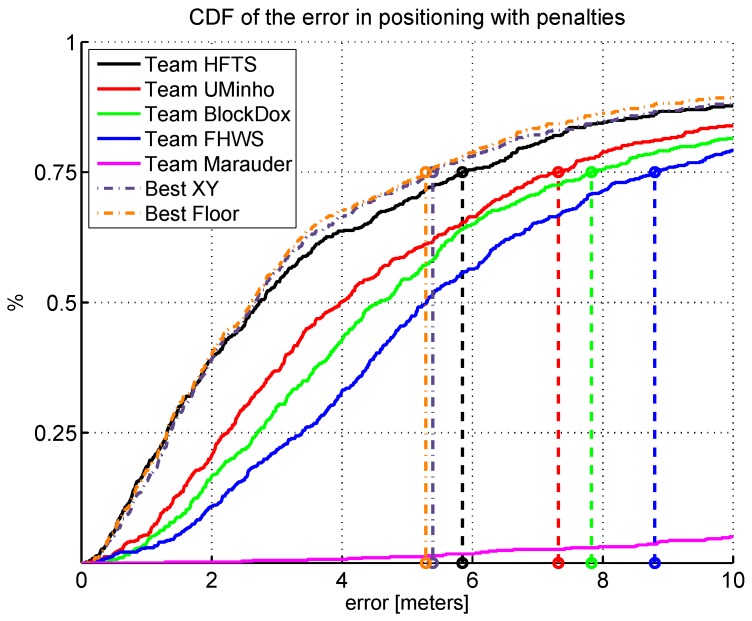
Cumulative distribution function (CDF) of the positioning error plus floor/building penalties obtained by each competitor.

Table 3 and Figure 3 summarize the competition results, with special emphasis on the main metric: the third quartile of the positioning error including penalties. Moreover, some collective and individual statistics on the evaluation logfiles are also shown in the table.

According to the main competition metric (the third quartile of the positioning error plus penalties), the HFTS team provided the best overall performing system, followed by the UMinho, BlockDox and FHWS teams. The Marauder team provided a huge positioning error. The mean error considering all logfiles and the cumulative distribution function (CDF) of the error in positioning shown in Figure 3 also ratify this ranking. Table 3 shows that the best floor identifications are provided by the UMinho and FHWS teams with a rate higher than 96%.

### Discussion on Performance

Table 3 also presents the mean error with penalties and floor identification rates for the different evaluation logfiles. The analysis of those results shows that the winning team (HFTS), provided excellent mean errors for logfiles 1, 4, 6 and 9 (UJITI and CAR Buildings), while the UMINHO and BlockDox teams provided the best results for logfiles 3 and 8 (UAH building collected with the Samsung S3 device). For the logfiles generated at the UAH building with the Samsung S4 device, HFTS provided the best results for logfile 5, whereas UMinho provided the best results for logfile 7.

We consider that better positioning may be obtained if we are able to combine the different solutions considering any synergy of the best proposals. For this reason, we have selected the best system for each validation trajectory. The best system has been selected according to two criteria: lowest positioning error without penalties (Best XY) and highest hit floor detection rate (Best Floor). For the former criterium (Best XY), the best systems were: HFTS (logfiles 01, 02, 04, 05, 06 and 09), UMinho (logfiles 03 and 07) and Block Dox (logfile 08). Please note that we selected them according to the positioning error without penalties; that is, the reason why the Block Dox system was chosen instead of UMinho system for logfile 08. For the latter criterium (Best Floor), it is important to mention that the best system was the one with highest floor detection rate, and, then, in the case of having multiple candidates, the lowest mean positioning error without penalties. For the Best Floor case, the best systems were: HFTS (logfiles 01, 02, 04, 05, 06 and 09) and UMinho (logfiles 03, 07 and 08). According to the results (Best XY and Best Floor), better accuracy and floor detection rate could be potentially obtained by combining different systems.

## 5. Discussion and Dataset Future Plans

We now discuss how to improve the datasets for future algorithm comparisons, according to the experience of creators of the data set and the feedback from its users (the competitors).

The reference logfiles included some prerecorded paths in the different buildings and the competitors had to apply their own strategies for training and validation. Some competitors suggested that including differentiated logfiles for training and validation would have been appreciated since they could have had a better overview of their systems accuracy and avoided programming errors in their systems. Moreover, they could have provided comparable results at the competition session.

After the competition, using the evaluation logfiles with ground truth (POSI headers), the Marauder team discovered that their Wi-Fi model was over-fitted because of their cross-validation strategy on small Wi-Fi fingerprinting data. According to their internal tests, their updated model had a score of 9.15 m on the competition metric and 88% floor hit rate.

Some competitors reported some long gaps without receiving Wi-Fi signals for some tests in the UAH building using the S3 phone. In particular, there were six gaps in logfile 03 and four gaps in logfile 05: all of them were longer than 60 s. These gaps in the Wi-Fi records were, probably, the cause for the large positioning errors reported by all of the competing systems in logfiles 03 and 08 (see Table 3) because there were many evaluation points which did not have any close WiFi fingerprint. Although some participants criticize this circumstance, this seems to be a natural behavior of a standard phone, probably due to low free memory space. The competition organizers consider this a realistic perturbation that the IPS developers must cope with. Thus, creating location systems robust to occasional measurement blackouts or bad sensor readings is, for us, an important topic of research.

According to the results shown in Table 3, the estimations for logfile02 are the ones with the worst floor identification rate. Some of the 91 evaluation points present in the logfile were located in the elevator (8) and walking up/down stairs (19). The organizers will study how to include them without highly affecting the main metrics.

Future repository logfiles will include Bluetooth low energy (BLE 4.0) RSSI information from detected devices. BLE is a relatively new technology with increasing commercial deployments. Future fingerprinting campaigns will include RSSI values from both Wi-Fi and BLE enabled devices. Moreover, as pointed out by the reviewers, we will provide the location of some Wi-Fi access points in order to consider those environments where their location is known.

In the 2016 edition, the data was mainly recorded by holding the phone on the hand in front of the actor’s chest. In future editions, it might be likely to include more challenging scenarios with swinging hand, pocket placement or phoning activities, resembling as close as possible the normal activity of people.

On the contrary, some competitors noted that the scenario provided in the current edition was interesting and challenging and suggested keeping it unchanged for future editions, since they have invested time and effort to adapt their systems to the existing format. An advantage of keeping the format is the possibility for new competitors to compare their results to those of previous editions. The organizers will balance this with introducing changes for the competition progressively, while maintaining the same format and spirit.

In order to ease the use of this data for competitors, more tools (such as parsers in common languages such as Python) will be developed and provided in our repository. An improved version of the Android Application used for data collection will be publicly available so the competitors can record information in their own labs and improve the training stages. We, the organizers, believe that the size of the community has the power to push innovation in the IPS field, so we plan to let people upload new generated logfiles in order to enrich the dataset with recordings collected from all over the world with their own devices (smartphones and tablets, mainly). This is our medium-term goal, which we think is the convenient way for creating a de-facto evaluation standard for smartphone-based indoor localization.

## 6. Conclusions

This paper shows an analysis of the off-site 2016 IPIN Competition and some important conclusions have been reached.

First, current smartphone technology provides an excellent tool for developing IPS based on the processing of signals from RF beacons, signals of opportunity and inertial data. Moreover, movement of user crowds may enable challenging applications as well as the creation of a huge de facto database for fair comparison of smartphone based IPS. Although the competition is over, the databases can be used to evaluate new developments from competitors or, even, new participants.

Second, diversity has been the key to the success of the competition. Using different environments, contexts, routes, and devices has led to a fair comparison that has not been attached to a particular environment, context or device.

Third, five systems, with significant different strategies, competed. The empirical results show that the best overall system may not be equally accurate in all environments. Appropriate selection or even fusion of diverse approaches may be the solution to obtain more robust systems.

Finally, the competition has been an excellent forum to meet experts who have proposed very different approaches to solve the same problem. The organizers considers that synergies and collaborations between groups have been reinforced after the competition, and discussion at the competition might lead to further advancements in indoor positioning systems.

## Figures and Tables

**Table 1:** Description of the training logfiles.

#	Building *	Route	Floors	Landmarks	Duration (s)	Smartphone
01	CAR	1	1	75	1257	S3
02	CAR	1	1	75	1260	S3mini
03	CAR	2	1	52	888	S3
04	CAR	2	1	52	887	S4
05	UAH	1	3	67	1101	S3
06	UAH	1	3	67	1101	S4
07	UAH	2	4	64	1192	S3
08	UAH	2	4	64	1188	S4
09	UAH	4	2	29	508	S3
10	UAH	4	2	29	508	S4
11	UJIUB	1	6	58	529	S3
12	UJIUB	1’	6	58	467	S3
13	UJIUB	2	6	59	397	S3
14	UJIUB	2’	6	59	375	S3
15	UJIUB	3	6	60	516	S3
16	UJITI	1	3	360	1134	GN5
17	UJITI	2	3	291	590	GN5

* See Figure 1; S3: Samsung Galaxy S3, Samsung, South Korea; S3mini: Samsung Galaxy S3 mini, Samsung, South Korea; S4: Samsung Galaxy S4, Samsung, South Korea; GN5: Google Nexus 5, LG Electronics, South Korea.

**Table 2:** Description of the evaluation logfiles.

#	Building *	Route	Floors	Landmarks	Duration (s)	Smartphone
01	UJITI	3	3	46	241	HW
02	UJIUB	4	6	91	730	S3
03	UAH	3	4	65	1476	S3
04	UJITI	4	3	75	430	SP
05	UAH	5	3	42	899	S4
06	CAR	3	1	76	1223	S3
07	UAH	3	4	65	1477	S4
08	UAH	5	3	42	899	S3
09	CAR	3	1	76	1223	S4

* See Figure 1; S3: Samsung Galaxy S3, Samsung, South Korea; S4: Samsung Galaxy S4, Samsung, South Korea; SP: Sony Xperia SP, Sony, Japan; HW: Huawei G630, Huawei, China.

**Table 3:** Overall results and main statistic value per logfile (best values in bold font). The 3rd quartile and mean errors are in meters, floor hit rates (flr in the table) are in percentage of correct estimates. The values for the ’all logfiles’ column correspond to the mean error and floor hit detection rate of the 578 validations points, and they do not correspond to the average of the nine logfiles.

		**All Logfiles**		**Logfile01**		**Logfile02**		**Logfile03**		**Logfile04**	
	**3rd Q.**	**Mean**	**flr**	**Mean**	**flr**	**Mean**	**flr**	**Mean**	**flr**	**Mean**	**flr**
**HFTS**	**5.85**	**5.76**	95.67%	**2.50**	**100%**	**5.16**	**89.01%**	18.27	84.62%	**2.03**	**100%**
UMinho	7.32	6.33	**96.54%**	4.03	97.83%	6.26	80.22%	**12.04**	**100%**	4.32	**100%**
BlockDox	7.83	7	92.73%	6.46	78.26%	5.61	87.91%	15.11	87.69%	3.66	**100%**
FHWS	8.8	8.23	96.02%	6	93.48%	7.8	78.02%	16.74	**100%**	5.94	**100%**
Marauder	40.9	32.6	51.38%	33.9	39.13%	22.57	32.97%	43.4	18.46%	36.42	42.67%
*Best XY*	5.39	4.94	96.19%	2.5	100%	5.16	89.01%	12.04	100%	2.03	100%
*Best Floor*	5.28	4.74	98.27%	2.5	100%	5.16	89.01%	12.04	100%	2.03	100%
		**Logfile05**		**Logfile06**		**Logfile07**		**Logfile08**		**Logfile09**	
		**Mean**	**flr**	**Mean**	**flr**	**Mean**	**flr**	**Mean**	**flr**	**Mean**	**flr**
**HFTS**		**4.49**	**100%**	**1.73**	**100%**	5.52	**100%**	13.2	88.10%	**2.23**	**100%**
UMinho		5.05	97.62%	5.76	**100%**	**4.45**	**100%**	**10.45**	**100%**	5.5	**100%**
BlockDox		7.33	90.48%	4.51	**100%**	7.36	92.31%	10.92	90.48%	5.17	**100%**
FHWS		8.18	**100%**	4.23	**100%**	6.32	**100%**	19.73	**100%**	4.39	**100%**
Marauder		25.83	35.71%	23.97	**100%**	57.38	26.15%	24.09	50%	26.7	**100%**
*Best XY*		4.49	100%	1.73	100%	4.45	100%	10.92	90.48%	2.23	100%
*Best Floor*		4.49	100%	1.73	100%	4.45	100%	10.45	100%	2.23	100%
